# Production of Verbascoside, Isoverbascoside and Phenolic Acids in Callus, Suspension, and Bioreactor Cultures of *Verbena officinalis* and Biological Properties of Biomass Extracts

**DOI:** 10.3390/molecules25235609

**Published:** 2020-11-28

**Authors:** Paweł Kubica, Agnieszka Szopa, Adam Kokotkiewicz, Natalizia Miceli, Maria Fernanda Taviano, Alessandro Maugeri, Santa Cirmi, Alicja Synowiec, Małgorzata Gniewosz, Hosam O. Elansary, Eman A. Mahmoud, Diaa O. El-Ansary, Omaima Nasif, Maria Luczkiewicz, Halina Ekiert

**Affiliations:** 1 Chair and Department of Pharmaceutical Botany, Faculty of Pharmacy, Jagiellonian University, Medical College, ul. Medyczna 9, 30-688 Kraków, Poland; p.kubica@uj.edu.pl; 2 Chair and Department of Pharmacognosy, Faculty of Pharmacy, Medical University of Gdansk, al. gen. J. Hallera 107, 80-416 Gdańsk, Poland; adamkokot@gumed.edu.pl (A.K.); mlucz@gumed.edu.pl (M.L.); 3 Department of Chemical, Biological, Pharmaceutical and Environmental Sciences, University of Messina, Viale Palatucci, 98168 Messina, Italy; nmiceli@unime.it (N.M.); mtaviano@unime.it (M.F.T.); amaugeri@unime.it (A.M.); scirmi@unime.it (S.C.); 4 Department of Food Biotechnology and Microbiology, Institute of Food Sciences, Warsaw University of Life Sciences—SGGW, ul. Nowoursynowska 159c, 02-776 Warsaw, Poland; alicja_synowiec@sggw.pl (A.S.); malgorzata_gniewosz@sggw.pl (M.G.); 5 Plant Production Department, College of Food and Agricultural Sciences, King Saud University, P.O. Box 2455, Riyadh 11451, Saudi Arabia; helansary@ksu.edu.sa; 6 Floriculture, Ornamental Horticulture, and Garden Design Department, Faculty of Agriculture (El-Shatby), Alexandria University, Alexandria 21545, Egypt; 7 Department of Geography, Environmental Management, and Energy Studies, University of Johannesburg, APK campus, Johannesburg 2006, South Africa; 8 Department of Food Industries, Damietta University, Damietta 34517, Egypt; emanmail2005@yahoo.com; 9 Precision Agriculture Laboratory, Department of Pomology, Faculty of Agriculture (El-Shatby), Alexandria University, Alexandria 21545, Egypt; diaaagri@hotmail.com; 10 Department of Physiology, College of Medicine, King Saud University (Medical City), King Khaled University Hospital, P.O. Box 2925, Riyadh 11461, Saudi Arabia; onasif@ksu.edu.sa

**Keywords:** vervain, in vitro cultures, phenylpropanoid glycosides, phenolics, antiproliferative effect, antioxidant activity, antibacterial effect

## Abstract

Callus, suspension and bioreactor cultures of *Verbena officinalis* were established, and optimized for biomass growth and production of phenylpropanoid glycosides, phenolic acids, flavonoids and iridoids. All types of cultures were maintained on/in the Murashige and Skoog (MS) media with 1 mg/L BAP and 1 mg/L NAA. The inoculum sizes were optimized in callus and suspension cultures. Moreover, the growth of the culture in two different types of bioreactors—a balloon bioreactor (BB) and a stirred-tank bioreactor (STB) was tested. In methanolic extracts from biomass of all types of in vitro cultures the presence of the same metabolites—verbascoside, isoverbascoside, and six phenolic acids: protocatechuic, chlorogenic, vanillic, caffeic, ferulic and rosmarinic acids was confirmed and quantified by the HPLC-DAD method. In the extracts from lyophilized culture media, no metabolites were found. The main metabolites in biomass extracts were verbascoside and isoverbascoside. Their maximum amounts in g/100 g DW (dry weight) in the tested types of cultures were as follow: 7.25 and 0.61 (callus), 7.06 and 0.48 (suspension), 7.69 and 0.31 (BB), 9.18 and 0.34 (STB). The amounts of phenolic acids were many times lower, max. total content reached of 26.90, 50.72, 19.88, and 36.78 mg/100 g DW, respectively. The highest content of verbascoside and also a high content of isoverbascoside obtained in STB (stirred-tank bioreactor) were 5.3 and 7.8 times higher than in extracts from overground parts of the parent plant. In the extracts from parent plant two iridoids—verbenalin and hastatoside, were also abundant. All investigated biomass extracts and the extracts from parent plant showed the antiproliferative, antioxidant and antibacterial activities. The strongest activities were documented for the cultures maintained in STB. We propose extracts from in vitro cultured biomass of vervain, especially from STB, as a rich source of bioactive metabolites with antiproliferative, antioxidant and antibacterial properties.

## 1. Introduction

*Verbena officinalis* L. (Verbenaceae)—vervain, is a perennial species, widespread throughout the globe, mainly in the temperate climate zone. This species has an established position in the European, North American, and East Asian medicine [1,2,3,4].

Since 2008, Verbena’s herb monograph has been included in the European Pharmacopoeia (6th ed.), which is obligatory for EU member states [5].

The verbena herb—blooming overground parts of the plant is characterized by the presence of several important groups of antioxidants–iridoids, phenylpropanoid glycosides, phenolic acids and flavonoids [6,7,8,9,10,11,12,13,14]. Iridoids are represented mainly by verbenalin, aucubin and hastatoside, while phenylpropanoid glycosides by verbascoside, isoverbascoside and eukovoside. In the group of phenolic acids, there are mainly ferulic acid, protocatechuic acid, and depsides—chlorogenic and rosmarinic acids, and dicaffeoylquinic acid derivatives. Flavonoids are represented by compounds commonly found in the plant kingdom—kaempferol, luteolin, and apigenin, however, some rare flavonoids like scutellarein and pedalitin are also found in the plant material. The plant produces an essential oil which is dominated by monoterpenoid components. Sterols, carbohydrates and numerous bioelements are also found in the herb [6,7,8,9,10,11,12,13,14].

In accordance with the requirements of the current European Pharmacopoeia (10th ed.), the raw material—the Verbena herb—should be standardized for verbenalin content (no less than 1.5% DW) [15].

Traditionally, the vervain herb is used as an antimicrobial, secretolytic, expectorant and diuretic agent. The advantage is also taken of its beneficial effect in the treatment of depression, insomnia and anxiety. In addition, the raw material is successfully used in the treatment of liver and gallbladder diseases [6,14,16].

The raw material has other scientifically-proven activities—antioxidant, antibacterial, antifungal, anti-inflammatory, analgesic, anticonvulsant, anxiolytic, antidepressant, sedative and hypnotic effects. It has also been proven to possess anti-cancer properties [14,17,18]. In addition, the vervain accelerates wound healing and has gastro-protective and insecticidal properties [14,19,20].

Because of the presence of various groups of antioxidants, a positive opinion regarding *V. officinalis* raw material was issued by the European Food Safety Authority (EFSA) [21]. The opinion states that the verbena herb can protect cells and tissues against damage caused by oxidative stress, and thus increases the body’s physiological resistance. Therefore, this raw material can and should be more commonly used in the production of functional foods and dietary supplements.

In addition, the species nowadays is recognized as a valuable cosmetic plant, mainly due to the presence of essential oil. According to the database of cosmetic raw materials kept by the European Commission (CosIng), *V. officinalis* is approved for use in the production of cosmetics [22].

The herb of vervain is characterized by high variability in chemical composition depending on its origin [23]. Therefore, the decision was made to establish in vitro cultures of this species. This is because they enable to control the environmental conditions and stimulate biosynthesis and accumulation of desired secondary metabolites [13]. In *V. officinalis*, the major compounds of interest are phenylpropanoid glycosides, represented mainly by verbascoside which was shown to exhibit anti-inflammatory, antioxidant, cytoprotective (including neuroprotective activity), antibacterial, antiviral and anti-androgen properties. Verbascoside also protects the skin from UV irradiation [24,25,26].

The basic factors that affect the accumulation of secondary metabolites in cell cultures are the composition of culture medium, including plant growth regulators (PGRs) composition, and lighting conditions. Our previous studies demonstrated a very clear effect of PGRs and lighting conditions on metabolite production in *V. officinalis* callus. The experiments proved that callus cells selectively accumulated large amounts of verbascoside (max. 2.4 g/100 g DW—light, 2.1 g/100 g DW—darkness) and selected phenolic acids (max. 46.02 mg/100 g DW, light—free compounds, max. 141.05 mg/100 g DW, light—bound compounds). The research enabled to select the best “production” medium which also provided good biomass growth: the MS medium variant containing 1 mg/L BAP (6-benzylaminopurine) and 1 mg/L IBA (indole-3-butyric acid). Using this medium, further research was conducted to investigate the effect of LED lights on metabolite accumulation [13,27].

The objective of the current research was to further optimize the conditions for growing agar callus cultures of *V. officinalis*, maintained on the MS medium supplemented with 1 mg/L BAP and 1 mg/L IBA, for improved production of verbascoside and isoverbascoside. This included the evaluation of the inoculum size on biomass growth and secondary metabolite production. Another objective of the work was to establish for the first-time suspension cultures of vervain which were optimized for inoculum size and production of bioactive metabolites. The next step of this research was the establishment, also for the first time, of vervain cultures grown in two different bioreactor constructions—stirred-tank bioreactor (STB) and balloon bioreactor (BB). The main aim was to scale up the biomass cultivation and production of biologically active metabolites. For comparative purpose, the concentrations of secondary metabolites in the herb extracts of parent plants were also determined.

Until now *V. officinalis* has been the object of biotechnological studies only connected with the elaboration of micropropagation protocols. Our team is the only one who established the in vitro cultures of this very valuable medicinal plant species to propose the biomass cultured in vitro as a rich source of bioactive metabolites independent of environmental conditions and environmental pollution.

In the second part of our research, a comparative evaluation of some biological activities of extracts obtained from biomasses grown in different in vitro systems and in the parent plant was performed. The toxicity of *V. officinalis* extracts was determined in vivo using the brine shrimp (*Artemia salina* Leach) lethality bioassay and the antiproliferative activity was assessed in vitro in the human neuroblastoma SH-SY-5Y cell line. The antioxidant potential of the extracts was evaluated through different in vitro methods: DPPH (1,1-diphenyl-2-picryl-hydrazyl), reducing power and ferrous ions (Fe^2+^) chelating activity assays. Moreover, the extracts were studied on antibacterial properties towards four Gram-positive and eight Gram-negative bacterial strains.

Our studies provide the first results documenting the potential therapeutic value of vervain biomass extracts from different types of in vitro cultures.

## 2. Results

### 2.1. Agar Callus Cultures

#### 2.1.1. Morphology, Inoculum Selection and Biomass Increases

Under the study, different inoculum sizes were tested (0.3, 0.6 and 0.9 g of fresh biomass for 30 mL of MS medium with 1 mg/L BAP and 1 mg/L IBA).

**Figure 1:**
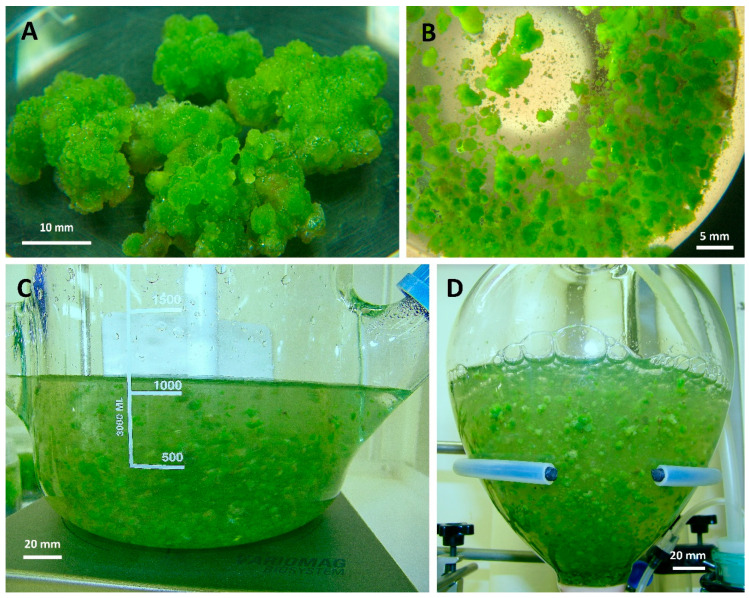
Different types of in vitro cultures of *Verbena officinalis* investigated in the study: (**A**), callus cultures (photograph without culture vessel); (**B**), suspension cultures grown in shake flasks (bottom view); (**C**), suspension cultures grown in stirred-tank bioreactor (STB); (**D**), suspension cultures grown in balloon bioreactor (BB). White bars indicate scale.

Macroscopic analysis of the appearance of callus cultures (embryogenic callus) showed the presence of globular embryos. During the experiment, they were observed to divide and form larger aggregates. The central part of the embryos had intensive green colour whereas the outer cells were parenchymatic and connected to neighbouring embryos through the central plaques. However, single embryos were not found. The tissue did not form cell clusters, which would favour further cytodifferentiation. In the outer parts, the biomass was dying—was characterized by a dark brown colour (Figure 1A).

**Figure 2:**
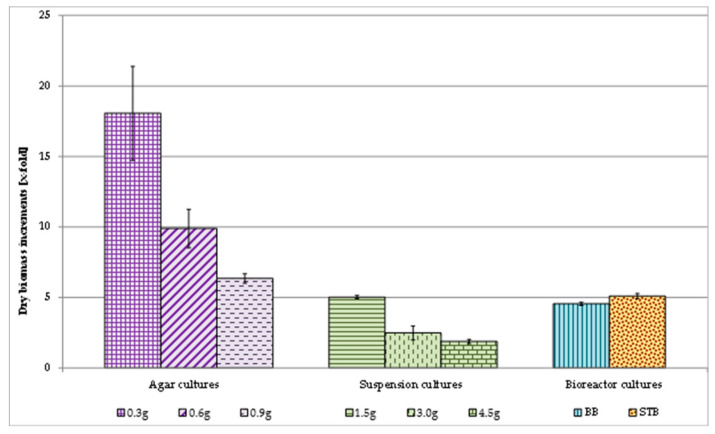
Dry biomass increments of *V. officinalis* in vitro cultures.

The biomass increments recorded during 2-week growth cycles varied depending on the inoculum to medium ratio. The values ranged from 6.66 to 16.77 times (fresh biomass) and from 6.34 to 18.06 times (dry biomass) (Figure 2). With the increase in inoculum size, a gradual decrease in biomass increments was observed, from 16.77–through 10.5–to 6.66-fold (fresh), and from 18.06–through 9.88–to 6.34-fold (dry biomass) (Figure 2).

#### 2.1.2. Phytochemical Analysis of Biomass Extracts

Two phenylpropanoid glycosides: verbascoside and isoverbascoside, and 6 phenolic acids: protocatechuic, chlorogenic, vanillic, caffeic, ferulic and rosmarinic acids, were found in the HPLC-DAD analyzed extracts from the biomass of agar cultures (Table 1 and Table 2).

The estimated compounds were not found in the analyzed extracts from lyophilized samples of the experimental media. Neither iridoids nor flavonoids were found in the extracts of biomass from callus and in the culture media.

In biomass extracts, the amounts of verbascoside varied moderately from 7154.93 (with 0.3 g of inoculum) to 7248.66 mg/100 g DW (with 0.9 g of inoculum). With 0.6 g of inoculum, the verbascoside content was almost identical to that obtained with 0.3 g of inoculum (7176.14 mg/100 g DW). The amounts of isoverbascoside were more varied, ranging from 442.81 (with 0.3 g of inoculum) through 528.17 (0.6 g of inoculum) to the maximum amount of 609.26 mg/100 g DW (0.9 g of inoculum) (Table 1). For the production of the two estimated phenylpropanoid glycosides, the determined optimal inoculum size was 0.9 g. With this amount of inocular biomass, the total amount of the two compounds was the highest (7857.92 mg/100 g DW).

The total amount of the estimated phenolic acids also varied depending on the amount of inoculum, from 19.42 mg/100 g DW (with the smallest inoculum of 0.3 g) through 26.90 mg/100 g DW (0.6 g) to 25.00 mg/100 g DW (0.9 g) (Table 2). The main phenolic acids were protocatechuic acid and ferulic acid. The amounts of these two compounds ranged from 6.91 to 9.58 mg/100 g DW, and from 4.91 to 7.35 mg/100 g DW, respectively. The maximum amounts of the other four phenolic acids, i.e., chlorogenic, vanillic, caffeic and rosmarinic acids, did not exceed 1.8, 1.3, 3.6 and 3.8 mg/100 g DW, respectively.

The sizes of inoculum that provide the best accumulation of the two groups of metabolites—phenylpropanoid glycosides and phenolic acids are 0.6 g and 0.9 g. It was with these amounts of inoculum that the high concentrations of both groups of compounds were obtained. However, the optimum amount of inocular biomass is 0.6 g, which provides both high production of secondary metabolites (7704.31 and 26.90 mg/100 g DW) and high dry biomass increases (9.88 times) (Table 1 and Table 2).

### 2.2. Suspension Cultures

#### 2.2.1. Morphology, Inoculum Selection and Biomass Increases 

As in the case of agar callus cultures, various inoculum sizes were tested during initiation of suspension cultures (1.5, 3.0 and 4.5 g of fresh biomass in 30 mL of medium). In the current work, *V. officinalis* suspension cultures were established for the first time (Figure 1B).

The studied embryogenic suspension cultures were characterized by fast growth. However, a tendency to necrosis has also been observed. Noticeable browning of the experimental media and tissue, observed already in the early days of the experiment, indicated either an advantage in the population of lysing cells or the secretion of phenolic compounds into the media.

During 2-week growth cycles, very different increments in both fresh and dry biomasses were recorded depending on the inoculum to volume-of-medium ratio. These increases ranged from 4.92 through 2.62 to 1.81 times (fresh biomass), and from 5.02 through 2.48 to 1.87 times (dry biomass) (Figure 2). As was the case with the agar cultures, with an increase in the size of inoculum there was a gradual decrease in biomass increments.

#### 2.2.2. Phytochemical Analysis of Biomass Extracts

The qualitative metabolite profile found in the analyzed extracts from the biomass of the suspension cultures was identical to that obtained for the agar cultures (Table 1 and Table 2). The presence of verbascoside and isoverbascoside, together with the same 6 phenolic acids was confirmed—these were protocatechuic, chlorogenic, vanillic, caffeic, ferulic and rosmarinic acids. As in the case of callus cultures, no iridoids and flavonoids were found in the analyzed samples, and also none of the metabolites was found in the extracts from the lyophilized media samples.

The analyses provided evidence of significant differences in the verbascoside content, dependent on the amount of inoculum. The concentrations ranged from 6288.95 (4.5 g of inoculum) to 7059.37 mg/100 g DW (3.0 g of inoculum). With the smallest inoculum size used (1.5 g of inoculum), the concentration of verbascoside was 6845.25 mg/100 g DW (Table 1). In the case of isoverbascoside, the analyses also showed large differences in its concentration. The amounts of this metabolite ranged from 264.36 mg/10 0g DW (1.5 g of inoculum) through 387.19 mg/100 g DW (3.0 g of inoculum) to the maximum of 477.25 mg/100 g DW (4.5 g of inoculum) (Table 1).

The total concentration of both phenylpropanoid glycosides ranged from 6766.21 mg/100 g DW (4.5 g of inoculum) through 7109.61 mg/100 g DW (1.5 g of inoculum), to the maximum of 7446.56 mg/100 g DW (3.0 g of inoculum). The amount of inoculum found to be optimal for the production of these two compounds was 3.0 g (Table 1).

The total phenolic acids content increased almost two-fold, depending on the inoculum size, from 28.09 mg/100 g DW (1.5 g of inoculum) through 49.46 mg/100 g DW (3.0 g) to a maximum of 50.72 mg/100 g DW (4.5 g) (Table 2). Quantitatively dominant metabolites were three acids—protocatechuic acid, ferulic acid and additionally rosmarinic acid. The concentrations of these compounds ranged from 2.09 to 10.64 mg/100 g DW, from 6.78 to 10.44 mg/100 g DW, and from 13.39 to 26.34 mg/100 g DW, respectively. The concentrations of the remaining three phenolic acids, i.e., chlorogenic, vanillic and caffeic acids, were lower, with maximum values of 1.25, 1.43 and 4.80 mg/100 g DW, respectively (Table 2).

The size of inoculum that should be considered as providing the best conditions for biomass growth and the accumulation of both groups of metabolites—phenylpropanoid glycosides and phenolic acids—is 1.5 g. With this inoculum size, the total concentration of verbascoside and isoverbascoside is high (7109.61 mg/100 g DW), and biomass increases are the highest—5.02-fold (dry biomass). However, the concentration of phenolic acids under these conditions is low (28.09 mg/100 g DW) (Table 1 and Table 2).

### 2.3. Bioreactor Cultures

#### 2.3.1. Morphology, Inoculum Selection and Biomass Increases

The suspension cultures were cultured for the first time in two types of bioreactors: a balloon bioreactor (BB) and a stirred-tank bioreactor (STB). In both cases, the working volume of the system was 1000 mL and the experiment duration was 2 weeks.

Macroscopic and microscopic observations revealed that biomass grown in BB was more viable than cells cultured in STB (Figure 1C,D). In the case of STB, during active growth, a faster browning of the growth medium and the presence of a suspension of broken cells accumulating in the vicinity of the bubbler was found. In the case of biomass incubated in BB, the medium did not turn brown during a 2-weeks growth cycle only showing slightly pearly opalescence (Figure 1C,D).

In both types of bioreactor, during the 2-week culture cycles, similar dry biomass increases of 4.55-fold (BB) and 5.09-fold (STB) were recorded (Figure 2).

#### 2.3.2. Phytochemical Analysis of Biomass Extracts

In the bioreactor cultures, the qualitative composition of secondary metabolites was found to be the same as in the agar and suspension cultures: the presence of verbascoside, isoverbascoside, and phenolic acids (protocatechuic, chlorogenic, vanillic, caffeic, ferulic and rosmarinic acids) was confirmed. None of the analyzed iridoids or flavonoids were found in the biomass. The estimated compounds were not found in the analyzed extracts from media lyophilizates.

High concentrations of verbascoside were found in biomass extracts—7685.00 mg/100 g DW (BB) and 9176.87 mg/100 g DW (STB) (Table 1). The amount obtained in the STB system was the highest obtained in the course of the study. The amounts of isoverbascoside obtained in BB and STB cultures were equal to 306.40 and 339.91 mg/100 g DW, respectively, and were markedly lower than those obtained in the agar callus cultures (max. 609.26 mg/100 g DW), and also lower than the maximum amounts found in the suspension cultures (477.25 mg/100 g DW) (Table 1).

The total amounts of verbascoside and isoverbascoside obtained in the STB were significantly higher than those in the BB (9516.79 and 7991.40 mg/100 g DW, respectively) (Table 1).

The confirmed total amounts of phenolic acids in the cultures grown in the BB were very low (19.88 mg/100 g DW) (Table 2). On the other hand, they were almost than twice as high (36.78 mg/100 g DW) in the cultures grown in the STB. In both types of bioreactors, the quantitatively dominant phenolic acids were the same as in the agar and/or suspension cultures, i.e., rosmarinic acid (10.22 and 14.87 mg/100 g DW), protocatechuic acid (5.07 and 9.68 mg/100 g DW), and ferulic acid (2.12 and 7.10 mg/100 g DW in BB and STB, respectively). The amount of total phenolic acids obtained in the STB bioreactor was higher than those of the agar callus cultures (19.42–26.90 mg/100 g DW) but lower than their maximum amount (50.72 mg/100 g DW) in the suspension cultures (Table 2).

### 2.4. Phytochemical Analysis of Parent Plant Material

**Figure 3:**
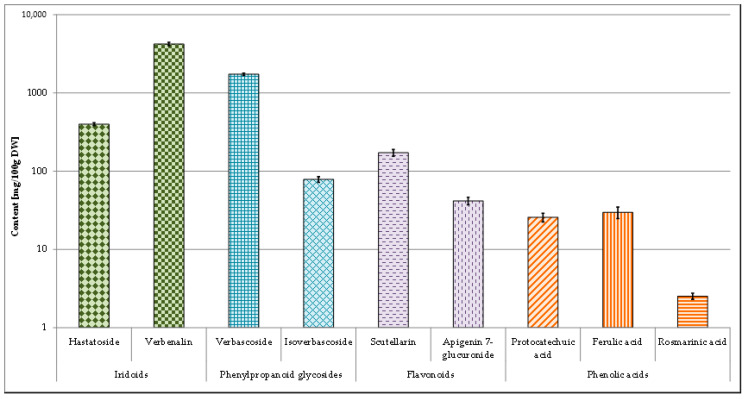
Iridoids, phenylpropanoid glycosides, flavonoids and phenolic acids contents (mg/100 g DW ± SD) in extracts of *V. officinalis* plant material.

The HPLC-DAD analysis of *V. officinalis* herb showed the presence of phenylpropanoid glycosides, phenolic acids, iridoids and flavonoids (Figure 3).

The analyzed herb extracts were found to contain a high amount of verbascoside (1728.97 mg/100 g DW) and a much lower concentration of isoverbascoside (78.34 mg/100 g DW). From the phenolic acids investigated, in extracts three compounds—protocatechuic acid (25.75 mg/100 g DW), ferulic acid (29.76 mg/100 g DW) and rosmarinic acid (2.53 mg/100 g DW) were estimated. Of iridoids, verbenalin and hastatoside were found in the analyzed herb extracts. The verbenalin content was very high—4210.88 mg/100 g DW. The concentration of hastatoside was lower—398.87 mg/100 g DW. Among flavonoids, scutellarin and apigenin 7-glucuronide were found in the analyzed herb extracts. The scutellarin content was 172.13 mg/100 g DW while apigenin 7-glucuronide content was about 4-fold lower—41.77 mg/100 g DW (Figure 3).

### 2.5. Artemia salina Leach Lethality Bioassay

The extract obtained from *V. officinalis* grown in vivo (*Vo*-in vivo) was found to be non-toxic for *A. salina*, with an LC_50_ > 1000 μg/mL; by contrast, the extracts obtained from in vitro biomasses resulted in toxic effects against brine shrimps. Based on Clarkson’s toxicity criterion, *V. officinalis* agar callus culture extract (*Vo*-C) showed low toxicity (LC_50_ = 698.67 μg/mL), whereas *V. officinalis* suspension culture extract (*Vo*-S) and *V. officinalis* stirred-tank bioreactor culture extract (*Vo*-STB) were moderately toxic, with an LC_50_ value of 479.34 μg/mL and 187 μg/mL, respectively [28].

### 2.6. Effect on SH-SY5Y Human Neuroblastoma Cell Proliferation

**Figure 4:**
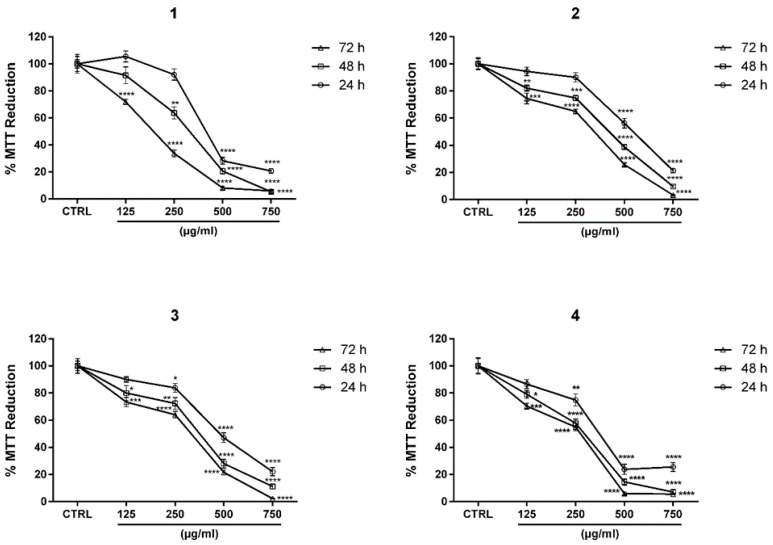
*V. officinalis* extracts reduce SH-SY5Y cell growth. The effect on cell proliferation was evaluated by 3-(4,5-dimethylthiazole-2-yl)-2,5-diphenyltetrazolium bromide (MTT) assay. SH-SY5Y cells were treated with increasing concentration (125–750 µg/mL) for 24, 48 and 72 h of each extract, as showed in figure. 1 = *Vo*-in vivo—*V. officinalis* parent plant grown in vivo-herb extract; 2 = *Vo*-C—*V. officinalis* callus culture extract; 3 = *Vo*-S—*V. officinalis* suspension culture extract; 4 = *Vo*-STB—*V. officinalis* stirred-tank bioreactor culture extract. Each value is the mean ± SEM of three different experiments performed in eight replicates. * *P* < 0.05 vs. ctrl, ** *P* < 0.01 vs. ctrl, *** *P* < 0.001 vs. ctrl and **** *P* < 0.0001 vs. ctrl.

Each extract was tested for its potential anti-proliferative activity on human neuroblastoma SH-SY5Y cell line, at concentrations ranging from 125 to 750 µg/mL, for 24–72 h. As shown in Figure 4, each extract was able to hinder SH-SY5Y cell growth, yet with different efficacy.

Considering the results in detail, both *Vo*-in vivo and *Vo*-STB significantly reduced cell viability by more than 70% at 500 µg/mL after 24 h of treatment, whereas the latter was the only one among the other tested extracts to reach an inhibition of more than 90% at 500 µg/mL after 72 h. *Vo*-C showed the weakest anti-proliferative activity among the others, reducing cell growth by more than 70% at 500 µg/mL and by 90% at 750 µg/mL after 72 h of treatment. *Vo*-S followed the same trend as *Vo*-C, reaching the 90% inhibition at the highest concentration after the longest exposure time; however, unlike *Vo*-C, it lowered growth rate by more than 70% at 500 µg/mL after 48 h of treatment.

These results were further confirmed by the extrapolation of IC_50_ values. In particular, *Vo*-STB showed the lowest IC_50_ value of 240.6 µg/mL at 72 h, followed by *Vo*-in vivo with an IC_50_ value of 275.1 µg/mL at 72 h. *Vo*-C and *Vo*-S showed IC_50_ values of 454.7 µg/mL and 419.9 µg/mL after 72 h of treatment, respectively.

### 2.7. Antioxidant Activity

The estimation of total phenolics contained in *V. officinalis* extracts was carried out using spectrophotometric analysis with Folin–Ciocalteu phenol reagent. As shown in Table 3, the total phenolic content varied from 126.55 ± 0.78 mg GAE/g extract (*Vo*-C) to 189.91 ± 2.93 mg GAE/g extract (*Vo*-S) and it decreased in the order: *Vo*- S > *Vo*-STB > *Vo*-in vivo > *Vo*-C.

**Figure 5:**
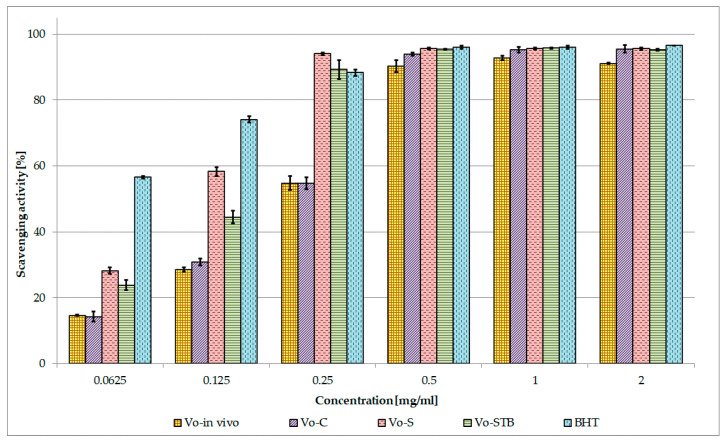
Free radical scavenging activity of *V. officinalis* extracts. Values are expressed as the mean ± SD (n = 3). *Vo*-in vivo—*V. officinalis* parent plant grown in vivo-herb extract; *Vo*-C—*V. officinalis* callus culture extract; *Vo*-S—*V. officinalis* suspension culture extract; *Vo*-STB—*V. officinalis* stirred-tank bioreactor culture extract; BHT—butylated hydroxytoluene.

The primary antioxidant efficacy of *V. officinalis* extracts was examined using the DPPH test and the reducing power assay; the secondary antioxidant properties were determined by measuring the chelating activity of the extracts. Furthermore, linear regression analysis was used to explore the relationship between the antioxidant activity of *V. officinalis* extracts and their phenolic content [29]. The results of the DPPH assay showed that all *V. officinalis* extracts exhibited strong radical scavenging ability (Figure 5). Among the extracts, *Vo*-S and *Vo*-STB were the most effective, as confirmed also by the IC_50_ values (0.110 mg/mL and 0.137 mg/mL); indeed, at the dose of 0.25 mg/mL both the extracts showed an activity superimposable to that of the standard BHT (88%), and much higher than that of *Vo*-in vivo and *Vo*-C extracts. At the concentration of 0.5 mg/mL, all the tested extracts reached their maximum effect, which was comparable to that of BHT (96%). Based on the comparison of IC_50_ values, the scavenging activity of extracts and standard decreased in the order: BHT > *Vo*-S > *Vo*-STB > *Vo*-C = *Vo*-in vivo (Table 3). A strong positive correlation was found between total phenolic content and DPPH radical scavenging activity (R^2^ = 0.9839).

**Figure 6:**
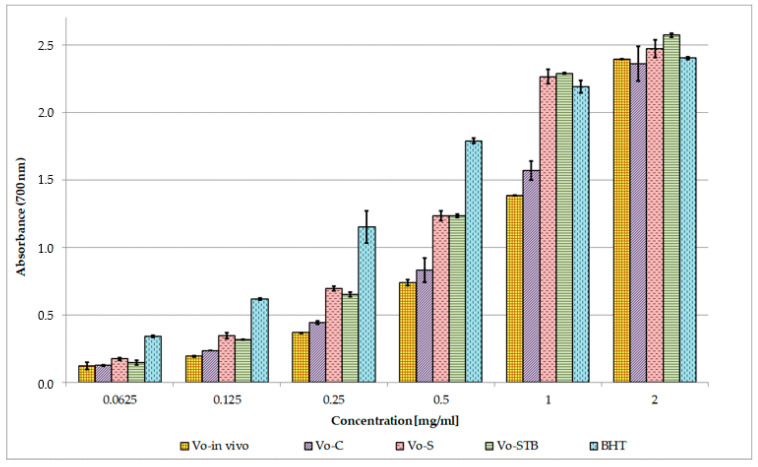
Reducing power of *V. officinalis* extracts. Values are expressed as the mean ± SD (n = 3). *Vo*-in vivo—*V. officinalis* parent plant grown in vivo-herb extract; *Vo*-C—*V. officinalis* callus culture extract; *Vo*-S—*V. officinalis* suspension culture extract; *Vo*-STB—*V. officinalis* stirred-tank bioreactor culture extract; BHT—butylated hydroxytoluene.

Figure 6 and Table 3 show the results of the reducing power assay. All the extracts displayed good reducing power, that increased with raising concentrations; in this test, *Vo*-S and *Vo*-STB also exhibited the strongest activity, as confirmed by the calculated ASE/mL values (ASE/mL = 1.762 and 1.779, respectively). At the concentration of 1 and 2 mg/mL, the reducing power of both the extracts was found to be slightly higher than that of BHT. Comparing the ASE/mL values, the reducing power of extracts and standard decreased in the following order: BHT > *Vo*-S = *Vo*-STB > *Vo*-C = *Vo*-in vivo. Finally, a strong positive correlation was found between total phenolic content and reducing power (R^2^ = 0.9629).

**Figure 7:**
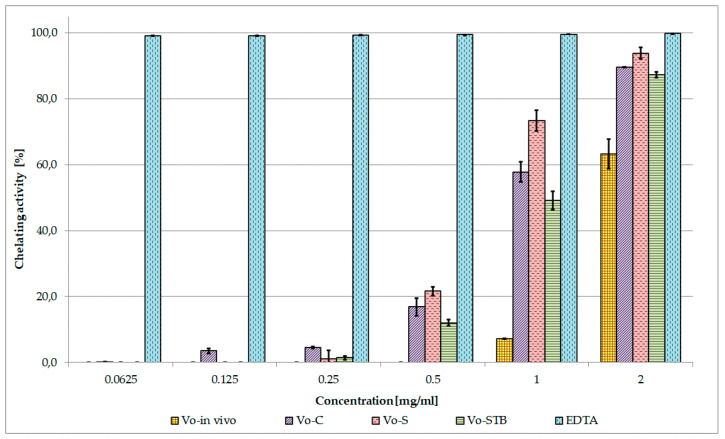
Ferrous ions (Fe^2+^) chelating activity of *V. officinalis* extracts. Values are expressed as the mean ± SD (n = 3). *Vo*-in vivo—*V. officinalis* parent plant grown in vivo-herb extract; *Vo*-C—*V. officinalis* callus culture extract; *Vo*-S—*V. officinalis* suspension culture extract; *Vo*-STB—*V. officinalis* stirred-tank bioreactor culture extract; EDTA—Ethylenediaminetetraacetic acid.

The results of the Fe^2+^ chelating activity assay showed that *Vo*-S, *Vo*-STB and *Vo*-C extracts interfered with the formation of the ferrous-ferrozine complex starting from the concentration of 0.5 mg/mL, displaying at the maximum tested dose (2 mg/mL) an activity slightly lower than that of the standard EDTA, whereas *Vo*-in vivo exhibited weak chelating effects only at the concentration of 1 and 2 mg/mL (Figure 7). According to the calculated IC_50_ values, *Vo*-S was found to be the most effective, followed by *Vo*-C (IC_50_ = 0.767 mg/mL and 0.908 mg/mL).

The metal chelating effect of the *V. officinalis* extracts and EDTA decreased in the order: EDTA > *Vo*-S > *Vo*-C > *Vo*-STB > *Vo*-in vivo (Table 3). Moreover, no correlation was found between metal chelating activity and total phenolic content (R^2^ = 0.2391).

### 2.8. Antibacterial Activity of Extracts

The MIC (Minimal Inhibitory Concentration) and MBC (Minimal Bactericidal Concentration) values of extracts obtained from *V. officinalis* were determined to evaluate their activity against selected pathogenic bacteria. *Vo—*in vivo, *Vo*-C, *Vo*-S and *Vo—*STB showed antibacterial activity against tested bacterial strains (Table 4). The *V. officinalis* extracts showed stronger bacteriostatic activity against Gram-positive bacteria (MIC 0.6−4.5 mg/mL), and lower in relation to Gram-negative bacteria (MIC 0.6–9.0 mg/mL). The extracts had similar bactericidal activity, and MBC values ranged from 0.6 to 18.0 mg/mL. The test strains had different sensitivity to the *V. officinalis* extracts. The most sensitive bacteria were *Y. enterocolitica*, *K. pneumoniae* (MIC/MBC 0.6–2.2 mg/mL) and *S. epidermidis* (MIC 0.6–1.1 mg/mL, MBC 0.6–9.0 mg/mL). Differences in the bacteriostatic and bactericidal activity of the extracts were also observed.

The bacteriostatic effect of *Vo*-in vivo was twice stronger against Gram-positive bacteria (MIC 0.6–2.2 mg/mL) than Gram-negative strains (MIC 1.1–4.5 mg/mL) and bactericidal activity was slightly lower. MBCs were in range 4.5–9.0 mg/ml and 4.5–18.0 mg/mL, respectively. The most sensitive strain was *S. epidermidis* (MIC/MBC 0.6/9.0 mg/mL), and the most resistant was *E. coli* (MIC/MBC 4.5/18 mg/mL).

*Vo*-C showed a weaker antibacterial effect than the other tested extracts. The growth of most Gram-positive strains was inhibited at the MIC of 9.0 mg/mL, and a bactericidal effect was observed at an MBC of 18.0 mg ml. *L. monocytogenes*, *E. aerogenes* and *E*. *coli* were more resistant to *Vo*-C.

*Vo*-S had weaker antibacterial activity compared to *Vo*-in vivo, but stronger than *Vo*-C especially against Gram-positive bacteria. *Vo*-S strongly inhibited the growth of *S. epidermidis* (MIC 0.6 mg/mL), but weakly *E. coli* (MIC 9.0 mg/mL).

*Vo*-STB had the best antibacterial effect among the investigated *V. officinalis* extracts. *Vo*-STB inhibited the growth of Gram-positive bacteria at MICs in the range of 0.6−2.2 mg /mL and Gram-negative at MICs in the range 0.6−4.5 mg/mL. In particular, strains of *S. epidermidis*, *Y. enterocolitica* and *K. pneumoniae* were more sensitive (MIC/MBC 0.6 mg/mL). *Vo*-STB also showed lower MBC values in relation to most strains compared *Vo*-in vivo, *Vo*-C and *Vo*-S.

Comparing the MIC/MBC values the antibacterial activity of extracts decreased in the following order: *Vo*-STB > *Vo*-in vivo > *Vo*-S > *Vo*-C.

## 3. Discussion

### 3.1. Biomass Increments

As demonstrated in numerous studies on cell cultures of higher plants, the selection of optimum inoculum size is crucial for obtaining high biomass yield [30]. In the *V. officinalis* agar callus cultures cultivated now on the best growth and production medium selected in our previous studies (MS, BAP—1 mg/L, IBA—1 mg/L), high, but strongly varied, increases in dry biomass were obtained, from 6.34 to 18.06 times, during 2-week culture cycles, depending on the ratio of the amount of inoculum to the volume of the medium. An increase in the inoculum size from 0.3 to 0.9 g resulted in biomass increments becoming gradually lower.

The biomass increases obtained in the current work were higher than the maximum increase obtained in our previous experiments with vervain (a 5.4-fold increase over 4-week growth cycles).

In the suspension cultures of *V. officinalis*, successfully established for the first time in the present work, also grown on the previously selected MS medium (containing 1 mg/L BAP and 1 mg/L IBA), the increases in biomass varied from 1.87 to 5.02 times (Figure 2), depending on the inoculum to volume-of-medium ratio. Satisfactory 5.02-fold increases were obtained during 2-week growth cycles with the smallest tested amount of inoculum (1.5 g per 30 mL of medium). Such biomass increments are satisfactory enough for suspension cultures.

After the suspension cultures of *V. officinalis* had been established, attempts were made to introduce them to bioreactor cultivation which is a crucial step in scale-up operations. In the current work, two bioreactors were employed: BB (balloon bioreactor) and STB (stirred-tank bioreactor). Both of these bioreactor types have been commonly used for plant cell cultivation, in the laboratory and on an industrial scale [31]. In the bioreactor cultures established by us, cultivated in BB and STB, the obtained increases in biomass during 2-week cycles were comparable (4.55 and 5.09 times) and satisfactory enough (Figure 2).

### 3.2. Phytochemical Analysis

Phytochemical analysis of herb was carried out to make a reliable assessment of the biotechnological research results.

The phytochemical analyses performed by us have demonstrated that biosynthetic profile of cells from in vitro cultures is different as compared to those of parent plant grown in vivo. Iridoids (verbenalin and hastatoside) dominate quantitatively in the herb of the plant. Quite large amounts of phenylpropanoid glycosides (verbascoside and isoverbascoside) and moderate amounts of phenolic acids and flavonoids were also found.

The secondary metabolism pathways that are dominant in the cells of in vitro cultured biomass are shikimate and cinnamate pathways leading to the formation of verbascoside and, to a lesser extent, isoverbascoside. Cell culture extracts were also found to have a phenolic acid profile comparable to that of the parent plant grown in vivo. The maximum amounts of phenylpropanoid glycosides obtained in cell cultures exceeded the values recorded for parent plant material. These results suggest that tissue differentiation in *Verbena* does not favour the accumulation of verbascoside and isoverbascoside. On the other hand, iridoids were absent in embryogenic calli and suspension cultures of vervain. These observations are in agreement with other studies. Sesterhenn et al. [32] demonstrated that callus cultures of *Scrophularia nodosa* were devoid of iridoids which are otherwise abundant in organized biomass of the species. Another example is the study by Grąbkowska et al. [33] which showed that tissue dedifferentiation in *Harpagophytum procumbens* in vitro cultures is positively correlated with verbascoside content and negatively correlated with the production of iridoids. The ability of non-organized cultures to accumulate verbascoside was also documented in the study on callus and suspension cultures of *Scrophularia striata* [34].

As revealed by HPLC analyses of media samples, all of the synthesized metabolites in *V. officinalis* suspension and bioreactor cultures were stored intracellularly which is a common attribute of plant in vitro cultures [31].

In the present work, high amounts of verbascoside were obtained in the agar callus cultures (max. 7248.66 mg/100 g DW), suspension cultures (max. 7059.37 mg/100 g DW), BB bioreactor (max. 7685.00 mg/100 g DW), and extremely high in the STB bioreactor (max. 9176.87 mg/100 g DW). Cultures were maintained in 14 days growth cycles which gives us the specific productivity 120.49, 74.36, 104.39 and 115.20 mg/L/day, respectively (Table 1). The amounts of this metabolite were, respectively, 3.2, 3.1, 3,4 and 4.1 times higher than its maximum concentration in the soil-grown parent plant (2263.85 mg/100 g DW). The amounts of isoverbascoside were also significant: max. 609.26 mg/100 g DW (specific productivity 10.13 mg/L/day) in the agar callus cultures, max. 477.25 mg/100 g DW (4.85 mg/L/day) in the suspension cultures, max. 306.40 mg/100 g DW (4.16 mg/L/day) in the BB bioreactor, and max. 339.91 mg/100 g DW (4.27 mg/L/day) in the STB bioreactor. The total amounts of verbascoside and isoverbascoside obtained in both suspension (7446.56 mg/100 g DW) and bioreactor (BB—7991.4 mg/100 g DW, STB—9516.79 mg/100 g DW) cultures are very high (Table 1).

High concentrations of both constituents were obtained within a short culture period (2 weeks), the established system can thus be considered as economically viable. The best results were obtained in the STB which belongs to the most commonly used bioreactor types [31]. The specific productivity in callus cultures is even higher than in STB cultures but obtaining biomass from bioreactor cultures is much easier and economically more profitable than obtaining it from agar cultures.

The in vitro system of *V. officinalis* established by us can be proposed as a rich source of phenylpropanoid glycosides, in particular verbascoside. The concentrations of this glycoside obtained are of practical interest, and what is most important, the highest amount was produced in bioreactor cultures (STB bioreactor—9.17 g/100 g DW) (Table 1).

Phenylpropanoid glycosides are often found in in vitro cultures of various plants. The highest concentration of verbascoside among plants grown in in vitro conditions were observed in *Buddleja cordata* cell suspension cultures and reached 11.6 g/100 g DW [35]. By comparison, the maximum contents of verbascoside as the main component in suspension cultures of *Salvia vulgaris* and *Cistanche salsa* have been 16 g/100 g DW and 689 mg/L, respectively [36,37]. It has to be noted, however, that those high amounts were obtained only after feeding with suitable precursors.

In other cell cultures investigated, the concentrations of verbascoside are much lower than those obtained in our research but concentrations of isoverbascoside were higher. For instance, *Verbascum thapsus* callus cultures contain a maximum of 4.7 g/100 g DW verbascoside and 5.0 g/100 g DW isoverbascoside. Suspension cultures of this species reached 3.9 g/100 g DW verbascoside and 2.9 g/100 g DW isoverbascoside [38]. Callus tissue of *H. procumbens* reached a maximum level of 3.1 g/100 g DW verbascoside, but the concentration of isoverbascoside—0.7 g/100 g DW, was higher than in our research [33]. *Scutellaria alpina* cell suspension cultures reached maximal content of verbascoside equal to 2.7 g/100 g DW [39].

From literature reports, up to now, the highest verbascoside specific productivity—165 mg/L/day, was reached in suspension cultures of *Harpagophytum procumbens* grown in pulse-aerated column bioreactor. In our research, the highest specific productivity was also high and reached in BB and STB 104 and 115 mg/L/day, respectively (Table 1) [40].

High amounts of phenylpropanoid glycosides are also observed in transgenic root cultures of various plants. The maximum verbascoside amounts obtained by us, above 9.2 g/100 g DW (Table 1), are comparable with those obtained in *Paulownia tomentosa* hairy root cultures, (max. 9.5 g/100 g DW DW), and higher than in transformed roots of *Rehmannia glutinosa* (max. 1.69 g/100 g DW). Moreover, the highest concentration of isoverbascoside obtained in our research (0.61 g/100 g DW) is higher than in *R. glutinosa* transformed roots (0.35 g/100 g DW) [24,41,42]. In adventitious root cultures of *Castilleja tenuiflora*, the maximum level of verbascoside was 1.5 g/100 g DW and the maximum concentration of isoverbascoside was 3.7 g /100 g DW [43].

The amounts of verbascoside obtained by us are among the highest recorded in in vitro cultures. From a practical point of view, the significant amounts of verbascoside that we were able to obtain in undifferentiated biomass of vervain should be considered an unquestionable success.

It should be emphasized that verbascoside and the accompanying isoverbascoside are compounds with very important medicinal values. They exhibit antioxidant, anti-inflammatory, anti-proliferative, antiviral and antibacterial properties, among others [24,25,26].

To sum up, the best biomass growth increments in *V. officinalis* in vitro cultures we reached in agar callus cultures. Although, the biomass increments of suspension and bioreactor cultures were high, too.

In the all tested types of in vitro cultures, identified metabolites were the same. The cells cultured in vitro produced primarily verbascoside and isoverbascoside, and lower amounts of phenolic acids. The amounts of these metabolites varied between the types of tested cultures. Noteworthy is the extremely high production of verbascoside in STB. Moreover, in this system, isoverbasoside production was also high, and the accumulation of phenolic acids was sufficient. The biomass increments in this system were about 5 times in two weeks, which is very good result from a biotechnological point of view.

### 3.3. Biological Activities

The second part of our research was focused on the comparative investigation of the antiproliferative, antioxidant, and antibacterial activities of *V. officinalis* extracts obtained from in vitro grown biomass and from the parent plant. Nowadays, finding new candidate drugs from the plant kingdom is highly appealing since an ever-increasing number of people is turning to natural remedies to treat or at least to ameliorate the symptoms of many diseases. Among these, chronic pathologies, such as cancer or degenerative ones, represent the main target of interest since, during longer therapies, it is more likely to come across the unwanted side effects of synthetic drugs, that natural ones instead are less prone to have. Moreover, natural extracts have the advantage that they can be used as multitarget drugs that target different intracellular targets [44]. Several studies reported the importance of natural products for this purpose [45]. In this regard, our study emphasized the biological value of extracts rich in both verbascoside and isoverbascoside, indicating that there is a clear link between their high contents in in vitro grown biomass and their proven biological activities. Indeed, the *V. officinalis* extract with the highest levels of verbascoside, *Vo*-STB, among the others present in this study, showed the strongest anti-proliferative activity in SH-SY5Y cells, with IC_50_ values from 1.5 to 1.9-fold lower than those of the values for other two extracts (Figure 4). This is in line with the scientific literature, where verbascoside is widely acknowledged as an anti-cancer agent that acts following several mechanisms to achieve this outcome, like activating the apoptotic machinery, influencing cell cycle or interacting with damaged DNA [24,25,26,46].

The results of *A. salina* lethality bioassay indicated *Vo*-STB as the most promising cytotoxic extract, which is in agreement with experiments conducted on human cell culture.

The antioxidant potential of *V. officinalis* extracts was investigated and compared through three in vitro tests based on different mechanisms of action. Several literature reports indicate a linear correlation between antioxidant activity and phenolic content of plant extracts [29,47]. Therefore, linear regression analysis was used to explore this relationship for *V. officinalis* extracts.

Strong primary antioxidant properties were highlighted for all the extracts and a positive correlation between polyphenol content and both DPPH and reducing power assays was found. Among the tested extracts, *Vo*-S and *Vo*-STB showed a higher primary antioxidant capacity than that of the parent plant grown in vivo. Compared to *Vo*-in vivo extract, *Vo*-S and *Vo*-STB contain higher quantities of phenylpropanoid glycosides and quite a similar amount of phenolic acids. *Vo*-C contains a concentration of verbascoside and isoverbascoside superimposable to *Vo*-S but about half the amount of phenolic acids, which could explain the lower antioxidant efficacy observed for the extract. These findings suggest that the antioxidant ability of the extracts may be attributed both to the phenylpropanoid glycosides and to the phenolic acids, whose antioxidant properties have been previously demonstrated [48,49,50,51].

As for the secondary antioxidant properties, all the biomass extracts displayed higher chelating activity than that of *Vo*-in vivo, which does not seem to be related to the phenolic content. In fact, *Vo*-S, exhibiting the highest total phenolic content, showed the best chelating properties, followed by *Vo-C*, containing the lowest total phenolic concentration. The lack of correlation between chelating activity and phenolic content is in agreement with previous researches published by some of the authors [52,53] and indicates that the observed activity might depend also on non-phenolic chelators present in the extracts. However, the partial involvement of phenylpropanoid glycosides and phenolic acids, whose chelating capacities have been reported, cannot be excluded [54,55]. The greater activity observed for *Vo-C* in comparison to *Vo-STB* could be explained, at least in part, by the presence of higher amounts of isoverbascoside whose Fe^2+^ chelating activity was previously demonstrated to be 2-fold stronger than that of verbascoside [54], and of chlorogenic acid, a strong iron chelator [55].

The antibacterial potential of *V. officinalis* extracts was assessed by MIC/MBC determination (Table 4). All the extracts showed bacteriostatic and bactericidal activity, although the power of the effect was different. The strongest antimicrobial activity was found for *Vo*-STB and *Vo*-S, which is an agreement with the results of antioxidant assays and the highest content of total phenolics, verbascoside, isoverbascoside and phenolic acids in these extracts. The strong mode of action of verbascoside against *S. aureus* is based on the inhibition of protein synthesis [56]. The antibacterial activity of *V. officinalis* extracts has been the subject of several studies. Results of previous research are consistent with ours, indicating that the verbena herb extracts were more active against Gram-positive bacteria than Gram-negative bacteria. The Gram-negative bacteria *E. coli* and *K. pneumoniae* showed resistance toward *V. officinalis* extracts [57]. The high sensitivity of bacteria *Y. enterocolitica* to other plant extracts has already been confirmed by other authors. The MIC values of plant extracts for these bacteria were comparable to the values obtained for *S. aureus* [58,59]. The similar study performed by another team also showed differences in the antibacterial activity of ethanol extracts of *V. officinalis* prepared from various parts of the plant. Extracts from the stems showed, in general, higher antibacterial activity than leaf and root extracts. However, the extracts were shown to be active against bacterial strains that cause serious infections, including methicillin-resistant *S. aureus* strains and multidrug-resistant *S. typhi* [60].

The results of our biological activity studies suggest that verbascoside and isoverbascoside, present in high quantities in in vitro grown biomass of vervain, likely contribute to the observed effects. Antioxidant, antiproliferative and antibacterial activities of the examined extracts could be connected with the high production of verbascoside which is present in much higher amounts than other compounds. The strongest potential was highlighted for *Vo*-S and *Vo*-STB cultures which accumulated the highest amounts of this constituent [24].

## 4. Materials and Methods

### 4.1. Chemicals

Acetic acid, methanol, sodium chloride, and sucrose were purchased from Chembur (Piekary Śląskie, Poland), trifluoroacetic acid purchased from Honeywell Riedel-de Haen (Seelz, Germany). HPLC-grade methanol and acetonitrile were purchased in Merck (Darmstadt, Germany). Plant culture media components, plant growth regulators NAA (naphthalene-1-acetic acid), BAP (6-benzylaminopurine) and IBA (indole-3-butyric acid) and agar were purchased in Duchefa Biochemie (Haarlem, Netherlands). The following commercially available standards for HPLC analyses were used: verbascoside, isoverbascoside, verbenaline and hastatoside (ChromaDex^®^, Los Angeles, California, USA) and caffeic acid, chlorogenic acid, ferulic acid, protocatechuic acid, rosmarinic acid, vanillic acid (Sigma-Aldrich Co., Munich, Germany). Scutellarin, scutellarein and apigenin 7-glucuronide obtained from ChemFaces (Wuhan, Hubei, China). Bacterial culture media: nutrient agar (NA), Müller-Hinton Broth (MHB) and Müller-Hinton agar (MHA) were purchased in BTL (Łódź, Poland). Resazurin sodium was purchased from Sigma-Aldrich Co. (St. Louis, USA). Ferrous chloride (FeCl_2_) was obtained from Carlo Erba (Milan, Italy). Unless indicated otherwise all chemicals were purchased from Sigma-Aldrich (Milan, Italy).

### 4.2. Parent Plant Material

Aerial parts of *Verbena officinalis* L. (Verbenaceae) were harvested in Garden of Medicinal Plants, Faculty of Pharmacy, Jagiellonian University, Medical College, Kraków (Poland). Plants were collected during their flowering in July 2017. The collected plant material was air-dried at room temperature in darkness.

### 4.3. Initiation *of in vitro* Cultures 

*Verbena officinalis* L. callus cultures were established from leaf buds in 2014 from plants growing in the Garden of Medicinal Plants, Faculty of Pharmacy, Medical College, Jagiellonian University, Krakow (Poland). Initial cultures were established on solid agar medium (7.2 g/L) according to Murashige and Skoog (MS) with the addition of sucrose (30 g/L) and two PGRs: 1 mg/L BAP and 0.5 mg/L NAA. Cultures were grown under continuous artificial light (88 ± 8 mol × m^−2^ × s^−1^, Philips-Flora-TLD 35W/33 fluorescent lamps, Philips, France) at 25 ± 2 °C, and subcultured at 28-day intervals. The cultures served as a source of biomass for establishing experimental in vitro cultures—each time, the callus grew for 28 days was used as the inoculum [13].

### 4.4. Experimental in vitro Cultures

All experimental biomasses, including callus, suspension and bioreactor cultures, were maintained in a phytotron at 23 ± 1 °C, with a light intensity of 88 ± 8 mol × m^−2^ × s^−1^ (Philips-Flora-TLD 35W/33 fluorescent lamps, Philips, France). The length of the day and night—photoperiod (cyclical lighting—17/24 h) was regulated using an electric system.

In the experiment, all agar callus cultures inocular biomasses of 0.3, 0.6 and 0.9 g were used. The biomasses were placed into in vitro culture jars (Sigma-Aldrich) containing 30 mL MS medium with 1 mg/L BAP and 1 mg/L IBA solidified with agar (7.2 g/L) and supplemented with 30 g/L sucrose. Callus growth and morphology were observed over 14-day growth cycles (3 series, 10 samples each).

For the establishment of suspension cultures, the biomass propagated on agar medium was transferred (1.5, 3 and 4.5 g of callus) to the liquid medium by gently dispersing the biomass with a steel tweezer, to minimize mechanical damage of tissue. Biomasses were placed into 100 mL Erlenmeyer flasks containing 30 mL of MS medium with 1 mg/L BAP, 1 mg/L IBA and 30 g/L sucrose, and closed with silicone foam stoppers. The cultures were incubated on an orbital shaker (Innova 2300 Platform Shaker, New Brunswick Scientific, USA) for 14 days at 120 rpm (3 series, 10 samples each).

The two types of bioreactors were applied for experimental suspension cultures: commercially available stirred-tank bioreactor (STB) (Bellco Glass, Vineland, NJ, USA), and custom-made balloon bioreactor (BB) (Figure 1C,D). The details of the used STB were presented earlier [61]. In the current work, the system was operated at 33 rpm and continuous 0.5 *vvm* aeration. The design of the BB bioreactor used was similar to the BB system previously used by the authors [62]. In the present study, a larger vessel was employed (170 mm i.d., 300 mm height). Continuous aeration (0.5 *vvm*) was provided through silicone foam sparger.

Based on the results obtained at the stage of suspension cultures, both devices were operated at 50 g biomass/1000 mL medium ratio. The composition of the growth medium was the same as in the case of suspension cultures. The single growth cycle lasted 14 days and for each bioreactor type, the experiment was run in triplicate.

The biomasses from all tested types of in vitro cultures were separated from the growth medium (agitated and bioreactor cultures were vacuum filtrated using G4 glass funnel). The collected biomasses and media samples were lyophilized using GT 2 apparatus (Finn Aqua Santasolo-Sohlberg, Tuusula, Finland). Harvested herb was air-dried in room temperature.

### 4.5. Extraction

For analyses of phenylpropanoid glycosides, 0.30 g of lyophilized biomass/dried herb was extracted five times with 3 mL portions of methanol in an ultrasonic bath for 30 min and the extracts were centrifuged and after filtration through a syringe filter (Millex^®^GP, Millipore, 0.22 μm, Filter Unit) analyzed using HPLC-DAD method.

For analyses of phenolic acids, flavonoids and iridoids, 10 mL of each extract were dried in crystallizers, dissolved in 2 mL of methanol and after filtration through syringe filters, they were subjected to HPLC-DAD analysis.

Lyophilized samples of 20 mL media were dissolved in 1 mL of methanol, filtered and analyzed by HPLC.

For the biological investigations, lyophilized biomasses and dried herb were extracted five times with methanol in an ultrasonic bath (Polsonic 3, Poland) for 30 min. Extracts were centrifuged, filtered, placed in the crystallizers and evaporated to dryness at room temperature to obtain the following dry extracts: *V. officinalis* callus culture extract (*Vo*-C), *V. officinalis* suspension culture extract (*Vo*-S), *V. officinalis* stirred-tank bioreactor culture extract (*Vo*-STB)and *V. officinalis*, grown in vivo, herb extract (*Vo*-in vivo).

### 4.6. Chromatographic Analyses—HPLC-DAD

#### 4.6.1. Phenylpropanoid Glycosides and Iridoids

Chromatographic analysis of phenylpropanoid glycosides (verbascoside and isoverbascoside) and iridoids (verbenaline and hastatoside) was carried out using the previously developed HPLC-DAD method according to Schönbichler, et al. [63]. The used analytical column was Kinetex C-18 analytical column (150× 4.6 mm, 2.7 μm; Phenomenex, Torrance, California, USA). The mobile phase consisted of two solvents: 0,1% trifluoroacetic acid and acetonitrile (gradient program). Compounds were estimated using a DAD detector. The detection wavelength was set at 330 nm for phenylpropanoid glycosides and at 235 nm for iridoids. The detected compounds were identified by the method of addition and comparison with UV-DAD spectra and retention times (Rt) of commercial standards. The quantification was carried out based on calibration curves.

#### 4.6.2. Phenolic Acids and Flavonoids 

Chromatographic analysis of phenolic acids was carried out by the HPLC-DAD method according to Ellnain-Wojtaszek and Zgórka [64,65] using HPLC-DAD-system (Merck-Hitachi) and a Purospher RP-18e analytical column (4× 250 mm, 5 mL; Merck, Darmstadt, Germany). The mobile phase consisted of two solvents: methanol with 0.5% acetic acid (1:4 *v/v*) and methanol (gradient program). The detection wavelength was set at 254 nm. The identity of detected compounds was confirmed by the method of standard addition and the comparison of UV-DAD spectra and the Rt values.

### 4.7. Artemia salina Leach Lethality Bioassay

Determination of median lethal concentration (LC_50_) was carried out according to the method previously reported [66]. Ten brine shrimp larvae, taken 48 h after initiation of hatching in artificial seawater, were transferred to Petri dishes, and artificial seawater was added to obtain a final volume of 5 mL. Subsequently, the *V. officinalis* extracts (*Vo*-C, *Vo*-S, *Vo*-STB, and *Vo*-in vivo), diluted in artificial seawater, were added at the final concentrations of 10, 100, 500 and 1000 μg/mL. After 24 h of incubation at 25–28 °C, the surviving larvae were counted. The assay was carried out in triplicate, and LC_50_ values were determined by the Litchfield and Wilcoxon’s method. The extract was considered active if the LC_50_ was lower than 1000 μg/mL.

### 4.8. Effect on SH-SY5Y Human Neuroblastoma Cell Proliferation

The human neuroblastoma cell line SH-SY5Y was originally obtained from ATCC (Rockville, MD, USA), maintained and kept as described by Cirmi et al. [67]. Cell culture reagents were from Carlo Erba (Milan, Italy). For cell viability assays, stock solutions of the four extracts were prepared in DMSO at the highest concentration depending on each solubility. Aliquots were stored at −20°C and defrosted prior to the dilution in culture media. For each extract, the highest DMSO concentration was used as a control for the corresponding extract concentration.

The effect of *V. officinalis* extracts (*Vo*-C, *Vo*-S, *Vo*-STB, and *Vo*-in vivo) on the growth rate of SHSY5Y was assessed by 3-(4,5-dimethylthiazole-2-yl)-2,5-diphenyltetrazolium bromide (MTT) assay, as stated by Celano et al. [68]. Cells were seeded at a density of 5 × 10^3^ cell/well in 96-well plates and incubated overnight at 37 °C. Then, the medium was removed and replaced with fresh one containing extracts dilutions ranging from 125 to 750 µg/mL, along with a control (untreated cells) and solvent control (DMSO). After incubation for 24, 48 and 72 h at 37 °C, the medium was replaced with a fresh one containing MTT at a concentration of 0.5 mg/mL and incubated for further 4 h. Afterwards, MTT solution was removed and the formazan crystals derived from the metabolic activity of viable cells were solubilized in 100 µL of HCl/isopropanol 0.1 N and regularly shaken. The optical density was spectrophotometrically measured at 570 nm with a reference filter at 690 nm using a microplate reader (iMark™, Bio-Rad Laboratories, Milan, Italy). All experiments were performed in eight replicates for three times. Results are calculated as a percentage of viable cells compared to untreated cells and expressed as mean ± SEM.

### 4.9. Antioxidant Activity

#### 4.9.1. Determination of Total Phenolic Content

The total phenolic content of *V. officinalis* extracts (*Vo*-C, *Vo*-S, *Vo*-STB, and *Vo*-in vivo) was determined by the Folin-Ciocalteu method, using a calibration curve of gallic acid as a reference [69]. Briefly, 100 µL of each sample solution were mixed with 0.2 mL Folin–Ciocalteu reagent, 2 mL of H_2_O, and 1 mL of 15% Na_2_CO_3_, and the absorbance was measured at 765 nm, after 2 h incubation at room temperature, with a model UV-1601 spectrophotometer (Shimadzu, Milan, Italy). The total phenolics were estimated as gallic acid equivalent (GAE) and expressed in mg GAE/g extract ± standard deviation (SD). The data were obtained from the average of three determinations.

#### 4.9.2. Free Radical Scavenging Activity

The free radical scavenging activity of *V. officinalis* extracts (*Vo*-C, *Vo*-S, *Vo*-STB, and *Vo*-in vivo) was determined using the DPPH (1,1-diphenyl-2-picrylhydrazyl) method [69]. An aliquot (0.5 mL) of a methanol solution containing a different amount of each extract (0.0625–2 mg/mL) was added to 3 mL of daily prepared methanol DPPH solution (0.1 mM). The optical density change at 517 nm was measured, 20 min after the initial mixing, with a model UV-1601 spectrophotometer (Shimadzu). Butylated hydroxytoluene (BHT) was used as a reference.The results, obtained from the average of three independent experiments, are reported as mean radical scavenging activity percentage (%) ± SD and mean 50% inhibitory concentration (IC_50_) ± SD.

#### 4.9.3. Reducing Power Assay

The reducing power of *V. officinalis* extracts (*Vo*-C, *Vo*-S, *Vo*-STB, and *Vo*-in vivo) was evaluated by spectrophotometric detection of Fe^3+^→Fe^2+^ transformation method [69]. Different amounts of samples (0.0625–2 mg/mL) in 1 mL solvent were mixed with 2.5 mL of phosphate buffer (0.2 M, pH 6.6) and 2.5 mL of 1% potassium ferricyanide [K_3_Fe(CN)_6_]. The mixture was incubated at 50 °C for 20 min. The resulting solution was cooled rapidly, mixed with 2.5 mL of 10% trichloroacetic acid, and centrifuged at 3000 rpm for 10 min. The resulting supernatant (2.5 mL) was mixed with 2.5 mL of distilled water and 0.5 mL of 0.1% fresh ferric chloride (FeCl_3_), and the absorbance was measured at 700 nm after 10 min. Ascorbic acid and BHT were used as a reference. The results, obtained from the average of three independent experiments, are expressed as mean absorbance values ± SD and ascorbic acid equivalent (ASE/mL) ± SD.

#### 4.9.4. Ferrous Ions (Fe^2+^) Chelating Activity

The Fe^2+^ chelating activity of *V. officinalis* extracts (*Vo*-C, *Vo*-S, *Vo*-STB, and *Vo*-in vivo) was estimated by measuring the formation of the Fe^2+^-ferrozine complex, according to the method previously reported [69]. Briefly, different concentrations of each sample (0.0625–2 mg/mL) in 1 mL solvent were mixed with 0.5 mL of methanol and 0.05 mL of 2 mM FeCl_2_. The reaction was initiated by the addition of 0.1 mL of 5 mM ferrozine. The mixture was shaken vigorously and left standing at room temperature for 10 min, then the absorbance of the solutions was measured spectrophotometrically at 562 nm. Ethylenediaminetetraacetic acid (EDTA) was used as a reference. The results, obtained from the average of three independent experiments, are reported as mean inhibition of the Fe^2+^-ferrozine complex formation (%) ± SD and IC_50_ ± SD.

### 4.10. Antibacterial Activity

#### 4.10.1. Bacteria Strains and Preparation of Inoculum

Four strains of Gram-positive bacteria (*Staphylococcus epidermidis* ATCC 12228, *Staphylococcus aureus* ATCC 25923, *Bacillus cereus* ATCC 11778 and *Listeria monocytoge*nes NIPH—NIH 17/11) and eight strains of Gram-negative bacteria (*Yersinia enterocolitica* O3 NIPH—NIH 383/11, *Pseudomonas aeruginosa* ATCC 27853, *Klebsiella pneumoniae* ATCC 13883, *Proteus mirabilis* ATCC 35659, *Shigella sonnei* NIPH—NIHs, *Salmonella enterica* subsp. *enterica* serovar *enteritidis* ATCC 13076, *Enterobacter aerogenes* ATCC 13048 and *Escherichia coli* ATCC 25922) were tested. All strains originated from the American Type Culture Collection (ATCC, Manassas, VA, USA), clinical strains came from the National Institute of Public Health—National Institute of Hygiene (NIPH—NIH, Warsaw, Poland). All test strains are obligate or opportunistic foodborne pathogens. The bacterial strains were cultured on nutrient agar (NA) and incubated at 37 °C for 24 h. Bacterial inocula were prepared in 0.85% NaCl (*w/v*) to reach a population of approximately 1 × 10^8^ cfu/mL.

#### 4.10.2. MIC and MBC Determination of Extracts

MIC and MBC of extracts against the test strains were carried out by the method of serial micro dilutions [70,71]. Two series of dilutions of the extracts were prepared in Mueller-Hinton Broth (MHB) medium in the concentration range from 36.0 to 0.3 mg/mL. An inoculum was added to each well of the polystyrene 96-well plates, each of a volume of 250 µL so that the final number of cells was 5 × 10^5^ cfu/mL. A well without extract was chosen as a positive control. Negative control was the medium (without the addition of extracts) together with the tested bacteria. The plates were incubated at 37 °C for 20 h. Afterwards, 25 µL of resazurin at 0.02% (*m/v*) (filtered through a 22 µm sterile filter) was then added to each well and the plates were incubated at 37 °C for 2 h. Bacterial growth was assessed based on the change in resazurin colour from violet to pink [72] and compared with the control sample. MIC examination of extracts was repeated three times. The MIC value was defined as the lowest concentration of extract, in which no growth of bacteria was observed and expressed in mg/mL.

To determine MBC, 100 μL bacterial culture from each well showing no bacterial growth were re-inoculated onto Mueller–Hinton agar (MHA) medium, which were incubated at 37 °C for 24 h. The plates were checked for growth of colonies. MBC was defined as the lowest concentration of extract, which resulted in complete inhibition of bacterial growth and expressed in mg/mL.

### 4.11. Statistical Analysis

Statistical comparison of data was carried out by using one-way analysis of variance (ANOVA) followed by Tukey–Kramer multiple comparisons test (GraphPAD Prism Software for Science). *P*-values lower than 0.05 were considered statistically significant.

## 5. Conclusions 

Optimization of the best culture conditions for biomass growth and for the production of bioactive compounds in three types of vervain in vitro cultures (callus, suspension, bioreactors cultures) was performed. Very good results were obtained in suspension culture and bioreactors cultures (balloon bioreactor—BB and stirred-tank bioreactor—STB) both established for the first time.

The biological activities of the biomass extracts from different types of in vitro systems—antiproliferative, antioxidant and antibacterial properties, were also documented for the first time and they were greater compared to that of the parent plant grown in vivo.

Based on the results, the STB was selected as the best in vitro system for the increased production of phenylpropanoid glycosides. In addition, the extract obtained from biomass produced in STB displayed significant efficacy in all the biological activity tests carried out in this study. Our results suggest that cultures maintained in STB could be a valuable source of bioactive compounds alternative to the parent plant grown in vivo.

The obtained results have the applicable character. We propose *V. officinalis* bioreactor culture maintained in the STB as an efficient, rich potential source of verbascoside and isoverbascoside independent of environmental conditions. The obtained maximal quantities of verbascoside (9.18%) and additionally the high amount of isoverbascoside (0.34%) in STB are interesting from a practical point of view.

## Figures and Tables

**Table 1:** Phenylpropanoid glycosides accumulation (mg/100 g DW ± SD) and their specific productivity* (mg/L/day) in studied callus, suspension and bioreactor *V. officinalis* in vitro cultures.

			Verbascoside				Isoverbascoside				Total Content		
			Content			Specific Productivity	Content			Specific Productivity			
***Vo-C***	**Weight of inoculum**	**0.3 g**	7154.93	±	201.31 ^a^	105.31	442.81	±	8.95 ^a^	6.52	7597.74	±	210.26 ^a^
		**0.6 g**	7176.14	±	288.90 ^a^	105.39	528.17	±	20.08 ^b^	7.76	7704.31	±	308.98 ^a^
		**0.9 g**	7248.66	±	253.76 ^a^	120.49	609.26	±	22.48 ^c^	10.13	7857.92	±	276.24 ^a^
***Vo-S***		**1.5 g**	6845.25	±	441.38 ^b^	67.67	264.36	±	12.15 ^d^	2.61	7109.61	±	453.53 ^b^
		**3.0 g**	7059.37	±	34.62 ^b^	74.36	387.19	±	1.92 ^e^	4.08	7446.56	±	36.54 ^c^
		**4.5 g**	6288.95	±	193.62 ^c^	63.88	477.25	±	11.52 ^a^	4.85	6766.21	±	205.14 ^d^
***Vo-BB***			7685.00	±	500.39 ^d^	104.39	306.40	±	25.20 ^f^	4.16	7991.40	±	525.59 ^a^
***Vo-STB***			9176.87	±	172.34 ^e^	115.20	339.91	±	41.90 ^g^	4.27	9516.79	±	214.24 ^e^

$*\mathrm{specific}\mathrm{productivity}=\frac{(\mathrm{maximum concentration for each molecule in}\;\frac{mg}{100g}\mathrm{DW})\;\times \;\mathrm{cumulative biomass in}\;{\mathrm{gL}}^{-1}}{100\;g\;\mathrm{DW}\;\times \;\mathrm{number of days after which the maximum concentration of the certain molecule was reached}}$ Values are expressed as the mean ± SD (n = 3). *Vo*-C—*V. officinalis* callus culture extract; *Vo*-S—*V. officinalis* suspension culture extract; *Vo*-BB—*V. officinalis* balloon bioreactor culture extract; *Vo*-STB—*V. officinalis* stirred-tank bioreactor culture extract. ^a–g^ Different letters within the same column indicate significant differences between mean values (*P* < 0.05).

**Table 2:** Phenolic acids accumulation (mg/100 g DW ± SD) in studied callus, suspension and bioreactor *V. officinalis* in vitro cultures.

			Protocatechuic Acid			Chlorogenic Acid			Vanillic Acid			Caffeic Acid			Ferulic Acid			Rosmarinic Acid			Total Content		
***Vo-C***	**Weight of Inoculum**	0.3 g	6.91	±	0.62 ^a^	1.49	±	0.07 ^a^	1.28	±	0.02 ^a^	2.63	±	0.04 ^a^	4.91	±	0.02 ^a^	2.20	±	0.11 ^a^	19.42	±	0.85 ^a^
		0.6 g	9.58	±	0.02 ^b^	1.77	±	0.03 ^b^	1.28	±	0.01 ^a^	3.52	±	0.01 ^b^	7.35	±	0.03 ^b^	3.40	±	0.38 ^b^	26.90	±	0.45 ^b^
		0.9 g	9.41	±	0.48 ^b^	1.57	±	0.02 ^c^	1.20	±	0.02 ^a^	3.48	±	0.07 ^b^	5.56	±	0.04 ^c^	3.79	±	0.55 ^b^	25.00	±	1.17 ^b^
***Vo-S***		1.5 g	2.09	±	0.10 ^c^	0.12	±	0.01 ^d^	1.43	±	0.04 ^b^	4.28	±	0.22 ^c^	6.78	±	0.02 ^b^	13.39	±	2.01 ^c^	28.09	±	2.39 ^b^
		3.0 g	7.00	±	0.02 ^a^	0.92	±	0.02 ^e^	1.30	±	0.03 ^a^	4.09	±	0.10 ^c^	9.81	±	0.51 ^d^	26.34	±	1.22 ^d^	49.46	±	1.90 ^c^
		4.5 g	10.64	±	0.06 ^d^	1.25	±	0.04 ^f^	1.10	±	0.10 ^c^	4.80	±	0.06 ^d^	10.44	±	0.03 ^e^	22.49	±	0.39 ^e^	50.72	±	0.68 ^c^
***Vo-BB***			5.07	±	1.12 ^e^	0.28	±	0.10 ^g^	0.59	±	0.36 ^d^	1.61	±	0.79 ^e^	2.12	±	0.67 ^f^	10.22	±	2.47 ^f^	19.88	±	5.51 ^a^
***Vo-STB***			9.68	±	0.90 ^b^	0.73	±	0.09 ^e^	0.73	±	0.15 ^d^	3.68	±	0.16 ^b^	7.10	±	0.85 ^b^	14.87	±	2.74 ^c^	36.78	±	4.90 ^d^

Values are expressed as the mean ± SD (n = 3). *Vo*-C—*V. officinalis* callus culture extract; *Vo*-S—*V. officinalis* suspension culture extract; *Vo*-BB—*V. officinalis* balloon bioreactor culture extract; *Vo*-STB—*V. officinalis* stirred-tank bioreactor culture extract. ^a–g^ Different letters within the same column indicate significant differences between mean values (*P* < 0.05).

**Table 3:** Determination of total phenolic content (calculated as gallic acid), free radical scavenging activity (DPPH test), reducing power, and ferrous ions (Fe^2+^) chelating activity of *V. officinalis* extracts.

*V. officinalis* Extracts	Total Phenolics mg GAE/g Extract (DW)	DPPH Test IC_50_ (mg/mL)	Reducing Power Assay ASE/mL	Fe^2+^ Chelating Activity IC_50_ (mg/mL)
***Vo*-in vivo**	136.59 ± 2.84 ^a^	0.222 ± 0.008 ^a^	2.626 ± 0.067 ^a^	1.695 ± 0.039 ^a^
***Vo-C***	126.55 ± 0.78 ^b^	0.224 ± 0.012 ^a^	2.609 ± 0.314 ^a,b^	0.908 ± 0.042 ^b^
***Vo-S***	189.91 ± 2.93 ^c^	0.110 ± 0.020 ^b^	1.762 ± 0.329 ^b,c^	0.767 ± 0.250 ^c^
***Vo-STB***	179.19 ± 1.09 ^d^	0.137 ± 0.030 ^c^	1.779 ± 0.100 ^b,c^	1.031 ± 0.042 ^d^
**Standard**	-	BHT 0.0656 ± 0.008 ^d^	BHT 0.891 ± 0.622 ^d^	EDTA 0.0067 ± 0.0003 ^e^

Values are expressed as the mean ± SD (n = 3). *Vo*-in vivo—*V. officinalis* parent plant grown in vivo-herb extract; *Vo*-C—*V. officinalis* callus culture extract; *Vo*-S—*V. officinalis* suspension culture extract; *Vo*-STB—*V. officinalis* stirred-tank bioreactor culture extract; ASE—ascorbic acid equivalent; GAE—gallic acid equivalent; BHT—butylated hydroxytoluene; EDTA—Ethylenediaminetetraacetic acid. ^a–e^ Different letters within the same column indicate significant differences between mean values (*P* < 0.05).

**Table 4:** MIC and (MBC) values (mg/mL) of *V. officinalis* extracts.

*V. officinalis* Extract	Gram-Positive Bacteria					
	*S.* *epidermidis*	*S.* *aureus*	*B.* *cereus*	*L.* *monocytogenes*	*Y.* *enterocolitica*	*K.* *pneumoniae*
***Vo-in vivo***	0.6 (9.0)	1.1 (9.0)	2.2 (4.5)	2.2 (9.0)	1.1 (4.5)	1.1 (4.5)
***Vo-C***	1.1 (9.0)	2.2 (4.5)	4.5 (9.0)	4.5 (18.0)	2.2 (4.5)	2.2 (4.5)
***Vo-S***	0.6 (9.0)	2.2 (9.0)	4.5 (9.0)	4.5 (9.0)	1.1 (9.0)	1.1 (9.0)
***Vo-STB***	0.6 (0.6)	4.5 (4.5)	4.5 (4.5)	2.2 (2.2)	0.6 (0.6)	0.6 (0.6)
	**Gram−Negative Bacteria**					
	***Ps.*** ***aeruginosa***	***P.*** ***mirabilis***	***Sh.*** ***sonnei***	***S.*** ***enteritidis***	***E.*** ***aerogenes***	***E.*** ***coli***
***Vo-in vivo***	1.1 (9.0)	2.2 (9.0)	2.2 (4.5)	4.5 (9.0)	4.5 (9.0)	4.5 (18.0)
***Vo-C***	2.2 (18.0)	4.5 (9.0)	9.0 (9.0)	9.0 (9.0)	9.0 (18.0)	9.0 (18.0)
***Vo-S***	2.2 (9.0)	2.2 (9.0)	9.0 (18.0)	4.5 (9.0)	9.0 (9.0)	9.0 (18.0)
***Vo-STB***	2.2 (2.2)	2.2 (2.2)	4.5 (4.5)	4.5 (4.5)	4.5 (4.5)	4.5 (4.5)

MIC—Minimal Inhibitory Concentration, MBC—Minimal Bactericidal Concentration. *Vo*-in vivo—*V. officinalis* parent plant grown *in vivo*-herb extract; *Vo*-C—*V. officinalis* callus culture extract; *Vo*-S—*V. officinalis* suspension culture extract; *Vo*-STB—*V. officinalis* stirred-tank bioreactor culture extract.
